# Running Up Stairs

**Published:** 1868-02

**Authors:** 


### RUNNING UP STAIRS.

It is frequently of advantage to others, besides politicians, to know which way the wind blows ; domestically, it is of considerable practical importance, when a walk or a ride is contemplated ; in this case Pater Familias can run up to the top of the house with his mouth shut, taking two or three stairs at a stride, the result being, that when he reaches the roof, there will be an instinctive desire to draw a long breath, and forcibly too ; this sends the air to the remotest branches of the wind-pipe and to the air cells, distending them to their fullest capacity ; such running from cellar to roof, involving the climbing of several pairs of stairs, would very greatly promote lung development and would ward off consumption from multitudes of the narrow chested and sedentary ; such a feat performed at three regular times every day, together with some pumping operation, would cause a physical development of the chest in a few weeks, or months at most, which actual measurement would mathematically demonstrate ; having the advantage over gymnasiums and out-door rides or walks, in that they can be attended to every day, rain or shine, cold or hot, and without costing any money ; it is to be hoped that many an invalid and sedentary reader will note the suggestion and practice upon it. If, occasionally, you find yourself "up" with all this work and some time still on your hands, have a rocking chair in some large room, at the top of the house, all cozy, quiet and clean, and in some of the old familiar tunes of the village church of your childhood, sing by the hour, with an open mouth and a loud voice, not on a penny whistle pitch, the psalms and hymns and spiritual songs of Watts and Wesley, of David and Moses, and "Deuteronomy and all them;" sing away and rock away, and don't be afraid if your neighbors do think you are a Methodist; for if you are a live one, the angels won't be ashamed of you; but while you are singing, think what the words mean and let your heart go out in that meaning; think, too, of the many who used to sing these same songs with you, side by side in the same pew, but who can sing them no more now, for their lips are still in death and their sweet voices are hushed in the churchyard grave, to be heard no more until you join together in singing the hallelujahs of the skies.

